# Multimodal multilayer network centrality relates to executive functioning

**DOI:** 10.1162/netn_a_00284

**Published:** 2023-01-01

**Authors:** Lucas C. Breedt, Fernando A. N. Santos, Arjan Hillebrand, Liesbeth Reneman, Anne-Fleur van Rootselaar, Menno M. Schoonheim, Cornelis J. Stam, Anouk Ticheler, Betty M. Tijms, Dick J. Veltman, Chris Vriend, Margot J. Wagenmakers, Guido A. van Wingen, Jeroen J. G. Geurts, Anouk Schrantee, Linda Douw

**Affiliations:** Department of Anatomy and Neurosciences, Amsterdam UMC, Vrije Universiteit Amsterdam, Amsterdam Neuroscience, The Netherlands; Institute of Advanced Studies, University of Amsterdam, The Netherlands; Department of Clinical Neurophysiology and MEG Center, Amsterdam UMC, Vrije Universiteit Amsterdam, Amsterdam Neuroscience, The Netherlands; Department of Radiology and Nuclear Medicine, Amsterdam UMC, University of Amsterdam, Amsterdam Neuroscience, The Netherlands; Department of Neurology and Clinical Neurophysiology, Amsterdam UMC, University of Amsterdam, Amsterdam Neuroscience, The Netherlands; Department of Neurology, Amsterdam UMC, Vrije Universiteit Amsterdam, Amsterdam Neuroscience, The Netherlands; Alzheimer Center Amsterdam, Department of Neurology, Amsterdam Neuroscience, Amsterdam UMC, Vrije Universiteit Amsterdam, The Netherlands; Department of Psychiatry, Amsterdam UMC, Vrije Universiteit Amsterdam, Amsterdam Neuroscience, The Netherlands; GGZ in Geest Specialized Mental Health Care, Amsterdam, The Netherlands; Department of Psychiatry, Amsterdam UMC, University of Amsterdam, Amsterdam Neuroscience, The Netherlands

**Keywords:** Cognition, Graph theory, Functional connectivity, Structural connectivity, Multiplex networks, Minimum spanning tree

## Abstract

Executive functioning (EF) is a higher order cognitive process that is thought to depend on a network organization facilitating integration across subnetworks, in the context of which the central role of the fronto-parietal network (FPN) has been described across imaging and neurophysiological modalities. However, the potentially complementary unimodal information on the relevance of the FPN for EF has not yet been integrated. We employ a multilayer framework to allow for integration of different modalities into one ‘network of networks.’ We used diffusion MRI, resting-state functional MRI, MEG, and neuropsychological data obtained from 33 healthy adults to construct modality-specific single-layer networks as well as a single multilayer network per participant. We computed single-layer and multilayer eigenvector centrality of the FPN as a measure of integration in this network and examined their associations with EF. We found that higher multilayer FPN centrality, but not single-layer FPN centrality, was related to better EF. We did not find a statistically significant change in explained variance in EF when using the multilayer approach as compared to the single-layer measures. Overall, our results show the importance of FPN integration for EF and underline the promise of the multilayer framework toward better understanding cognitive functioning.

## Author Summary

Until now, the relationship between brain network topology and cognition has mostly been studied using isolated modal information (e.g., functional MRI or magnetoencephalography). Such isolated analyses ignore potentially complementary information. Here, we use multimodal imaging and neuropsychological data collected from healthy adults to demonstrate that increased centrality of the fronto-parietal network in a multilayer network is related to better executive functioning. We find no such relation for single-layer networks. These results show the importance of fronto-parietal network integration for executive functioning, as well as the value of a multilayer framework in network analyses of the brain.

## INTRODUCTION

A thread of network thinking runs through the history of cognition research. In 1983, Fodor introduced the ‘modularity of mind’ theory of cognition and behavior (Fodor, 1983). He posited that lower order processes of the mind are modular, with domain-specific modules operating independently without interacting with other modules. Contrastingly, he argued that higher order cognitive processes such as executive functioning (EF), which is thought to be the most complex and evolutionarily special cognitive domain (Ardila, 2008), are global rather than modular. Likewise, the evolution of neuroscience has led to a data-driven approach toward understanding how the brain governs such higher order cognition by studying the brain as a complex network through the framework of graph theory (Barabási, 2016; Bullmore & Sporns, 2009). Brain regions are thus represented as nodes, and the interactions between them as links.

Different modalities can be used to obtain these brain networks. Anatomically, diffusion magnetic resonance imaging (dMRI) maps the physical connections (i.e., white matter bundles) between the neural elements of the brain, yielding a structural network. Functionally, multiple imaging techniques can be used to observe brain activity. Resting-state functional magnetic resonance imaging (rsfMRI) detects variations in blood oxygenation as an indirect measure of neuronal activity at a high spatial resolution, and magnetoencephalography (MEG) provides a direct measure of the summed electromagnetic activity generated by groups of neurons. In both rsfMRI and MEG, statistical interdependencies between levels of activity in different areas of the brain are used as a measure for functional connectivity (Aertsen et al., 1989; Friston et al., 1993), yielding functional networks.

The organization of these structural and functional networks appears to be crucial for EF. Although the exact mechanisms underlying this cognitive function remain unknown (Jurado & Rosselli, 2007), EF appears to be highly reliant on network integration, that is, the interplay between specialized modules (Bullmore & Sporns, 2012). Key in facilitating this integration is the fronto-parietal network (FPN), a module that plays a crucial central role as a ‘connector’ within the brain network, having diverse connections to other modules of the brain (Bertolero et al., 2017). The network integration that is hypothetically happening in the individual brain regions that form the FPN can be characterized through network measures of centrality. Nodal centrality reflects the relative importance of a node within the network. Highly central regions are typically connected to many other regions, implying a pivotal role in the facilitation of network integration (Bertolero et al., 2017; van den Heuvel & Sporns, 2011). Indeed, a more central role of the FPN has been related to better EF in unimodal network studies that utilized dMRI (Caeyenberghs et al., 2016), rsfMRI (Cole et al., 2012; Takeuchi et al., 2015), or MEG (van Dellen et al., 2013).

However, in such unimodal network studies the different aspects of the brain network, for example, structural and functional, are only studied in isolation, while we know from other types of complex networks that network structure and different types of functional dynamics occurring on top of it jointly and synergistically determine system behavior (Boccaletti et al., 2006, 2014; Cardillo et al., 2013; Zanin, 2015). In the brain, it remains unclear exactly how the integration between these network aspects relates to EF. Nevertheless, the interplay between structural and functional connectivity has been shown to be nontrivial, suggesting both should be considered simultaneously (Damoiseaux & Greicius, 2009; Park & Friston, 2013; Stam et al., 2016). Moreover, in the case of networks based on MEG data, the broadband signal is often filtered into canonical frequency bands, and network analysis is performed for each frequency band separately, but the different imaging modalities and frequency bands each yield unique and even complementary information that should perhaps not be considered in isolation. Unimodal networks are thus limited representations of the essentially multimodal brain network (Garcés et al., 2016; Mandke et al., 2018; Zanin, 2015), but until recently we lacked the appropriate tools to integrate multiple modalities into a single network representation.

**Figure 1.**
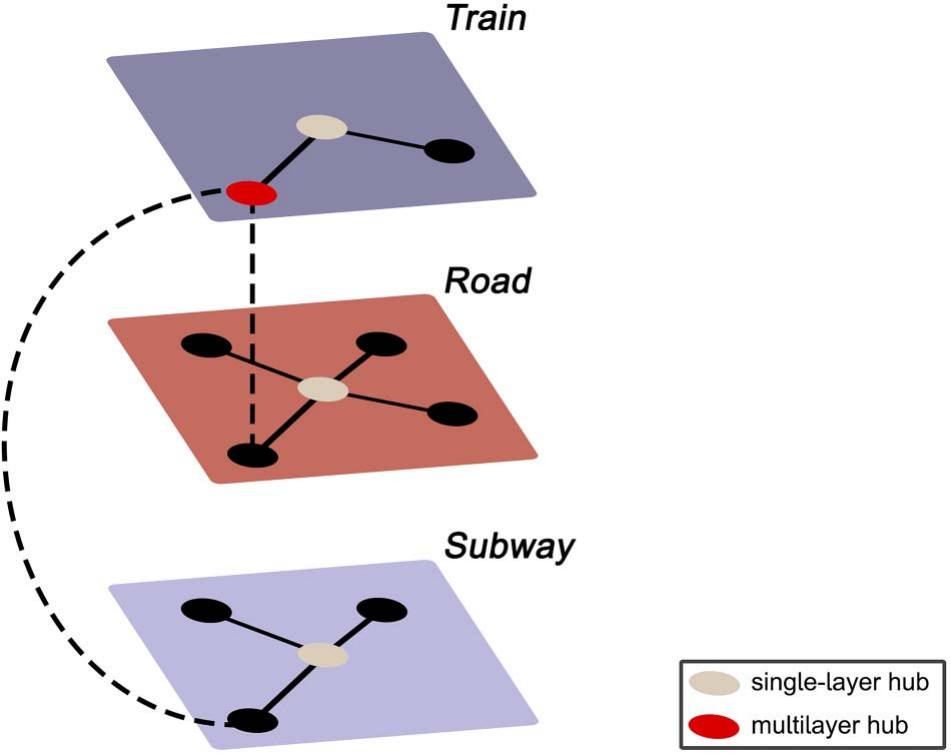
Example of a multilayer transport network, encoding information about train, subway, and road connectivity, and the interlayer links between them. Suppose there is a sudden increase in commuters in the subway network in the absence of any delayed or cancelled subway cars or suspended subway stations. Considering the single-layer subway network in isolation, the observed spike in commuters would seem inexplicable, as the properties of the subway network (i.e., the links and nodes) are unaltered. However, observing the entire transport system might reveal severe delays in the train network, forcing people who usually commute by train to now use the subway, thus leading to an increase in commuters in the subway network. Likewise, consider the red node in the train network. From a single-layer perspective, this is a peripheral station that is of little importance to the transport system. However, the multilayer perspective reveals this to be the only location where all three modes of transport connect, and the seemingly peripheral station thus plays a significant integrative role in the transportation network—a property that would have remained unnoticed without incorporating all the layers of the system.

Multilayer network analysis is a newly developed mathematical framework that enables this integration and allows for analysis of multimodal data (Boccaletti et al., 2014; De Domenico et al., 2013; Kivelä et al., 2014). A multilayer network is a ‘network of networks,’ comprised of multiple interconnected layers, each characterizing a different aspect of the same system. Figure 1 illustrates the concept of multilayer networks using the analogy of a commuter network. Although the framework of multilayer networks is relatively new in the field of neuroscience, promising results have already been reported. Multilayer analysis of dMRI and fMRI networks of healthy participants confirmed the synergistic nature of the structure and function of the brain network (Battiston et al., 2017). Further relevance of multilayer analysis has been shown in clinical studies: multilayer connectivity differences were reported between patients with schizophrenia and healthy controls, and these differences were related to symptom severity (Brookes et al., 2016). Moreover, a study in schizophrenia and another study in Alzheimer’s disease suggested that multilayer centrality could outperform single-layer measures to distinguish cases from healthy controls (De Domenico et al., 2016; Guillon et al., 2017). Additionally, an MEG study used nodal centrality metrics to identify brain regions that were vulnerable in patients with Alzheimer’s disease compared to healthy controls and found that such regions could only be detected using a multilayer approach. Even more relevant to our work, this vulnerability of central regions in the multilayer network was related to cognitive dysfunction (Yu et al., 2017). Multilayer network analysis can thus contribute to a better understanding of the relation between the FPN and EF.

Here, we used multimodal data to assess the association between FPN centrality and EF in healthy participants and statistically explored the potential added value of a multilayer framework over a single-layer framework. We (a) hypothesized a positive association between FPN centrality of both the single- and multilayer networks, which could already indicate that the multilayer approach is useful to integrate multimodal data toward investigating executive functioning. Moreover, we (b) aimed to test whether multilayer centrality supersedes its single-layer equivalents in explaining individual differences in EF statistically, through a significant change in explained variance.

## METHODS

### Participants

This study was preregistered in the International Clinical Trials Registry Platform under trial ID NTR7510. Thirty-nine (39) healthy participants were prospectively recruited for this specific study through an online platform, Hersenonderzoek.nl (www.hersenonderzoek.nl), where volunteers can register for participation in neuroscience studies. Participants were selected based on the following inclusion criteria: (a) age between 20 and 70 years old, (b) native Dutch speaker, and (c) able to provide written informed consent. The following exclusion criteria were used: (a) history of neurological or psychiatric disease, (b) current and regular use of centrally acting drugs, and (c) presence of contra-indications for MRI or MEG. Participants were asked not to ingest any caffeine or alcohol on the testing days. Approval was obtained from the VU University Medical Center Medical Ethical Committee, and all subjects provided written informed consent prior to participation.

### Neuropsychological Evaluation

Participants underwent an extensive customized neuropsychological test battery, consisting of the Dutch version of Rey’s Auditory Verbal Learning Test (van den Burg et al., 1985), the Concept Shifting Test (CST; van der Elst et al., 2006a), the Memory Comparison Test (van der Elst et al., 2007), the Stroop Color-Word Test (SCWT; Hammes, 1978), the Location Learning Test (LLT; Bucks & Willison, 1997), the Categorical Word Fluency Test (Mulder et al., 2006), and the Letter-Digit Modalities Test (van der Elst et al., 2006b). We used (subscores on) three of these tests to assess EF. The first test we used was the CST, where the participant was shown 16 small circles, grouped in a large circle, containing either digits (CST part A), letters (CST part B), or both digits and letters (CST part C). These circles needed to be crossed out in ascending order in part A, in alphabetical order in part B, and in alternating order (digit-letter) in part C. The participant was asked to perform the test as quickly as possible without making mistakes. Additionally, to correct for motor speed, a null-condition with empty circles (CST zero) was carried out three times. The second test we used was the SCWT, where the participant was asked to read four different cards. On the first card, names of colors—red, green, yellow, and blue—were printed in black ink. On the second card, rectangles were printed in these same colors. On the third card, the names of the colors were printed in an incongruent color ink; for example, the word ‘red’ was printed in yellow ink, and the participant was asked to read the color of the ink and ignore the word. The fourth card was identical to the third card, but several words were circled. For these circled words, the participant was asked to read the word itself instead of the color of the ink. The third test we used was the Word Fluency Test, where the participant was asked to name as many words in the category ‘animals’ as possible within 60 seconds.

We used validated norms of the CST, SCWT, and Word Fluency Test to transform raw scores into *z*-scores relative to a comparable healthy population. Briefly, raw CST scores were adjusted for age only, as no effects of gender or age squared were present in the normative sample of 1,794 Dutch adults (van der Elst et al., 2006a). Raw SCWT scores were corrected for age, age squared, and education (classified according to the Dutch Verhage system (Verhage, 1964), which ranges from level 1 [less than 6 years of primary education] to level 7 [university degree]) (Schmand et al., 2012). Raw scores on the Word Fluency Test were corrected for age and education, as there were no effects of age squared in the normative sample (Schmand et al., 2012). EF was defined as the average of *z*-scores for Word Fluency, Stroop-interference (time to complete card 3 corrected for the time to complete card 2), and CST-shift (time to complete card C minus the average time to complete cards A and B, adjusted for time to complete CST zero).

### Magnetic Resonance Imaging

MRI data were obtained using a 3T MRI system (Philips Ingenia CX) with a 32-channel receive-only head coil at the Spinoza Centre for Neuroimaging in Amsterdam, The Netherlands. A high-resolution 3D T1-weighted image was collected with a magnetization-prepared rapid acquisition with gradient echo (MPRAGE; TR = 8.1 ms, TE = 3.7 ms, flip angle = 8°, voxel dimensions = 1 mm^3^ isotropic). This anatomical scan was registered to MNI space through linear registration with nearest-neighbor interpolation, and was used for co-registration and normalization of all other modalities (dMRI, fMRI, and MEG) to the same space.

#### Diffusion MRI.

Diffusion MRI was collected with diffusion weightings of b = 1,000 and 2,000 s/mm^2^ applied in 29 and 59 directions, respectively, along with 9 nondiffusion weighted (b = 0 s/mm^2^) volumes using a multiband sequence (MultiBand SENSE factor = 2, TR = 4.7 s, TE = 95 ms, flip angle = 90°, voxel dimensions = 2 mm^3^ isotropic, no interslice gap). In addition, two scans with opposite phase encoding directions were collected for blip-up blip-down distortion correction using FSL topup (Andersson et al., 2003). Structural connectomes were constructed by performing probabilistic anatomically-constrained tractography (ACT) (Smith et al., 2012) in MRtrix3 (Tournier et al., 2019). A tissue response function was estimated from the preprocessed and bias field corrected dMRI data using the multishell multitissue five-tissue-type algorithm (msmt_5tt). Subsequently, the fiber orientation distribution for each voxel was determined by performing multishell multitissue constrained spherical deconvolution (MSMT-CSD) (Jeurissen et al., 2014). ACT was performed by randomly seeding 100 million fibers within the white matter to construct a tractogram, and spherical-deconvolution informed filtering of tractograms (SIFT, SIFT2 method in MRtrix3) (Smith et al., 2015) was then performed to improve the accuracy of the reconstructed streamlines and reduce false positives. For every participant, their respective 3D T1-weighted image was used to parcellate the brain into 210 cortical Brainnetome atlas (BNA) (Fan et al., 2016) regions. We then used this parcellation to convert the tractogram to a structural network, where weighted edges represented the sum of all streamlines leading to and from all voxels within two brain regions.

#### Resting-state functional MRI.

Resting-state fMRI was collected using a multiband sequence (MultiBand SENSE factor = 2, TR = 1.52 s, TE = 30 ms, flip angle = 70°, voxel size = 2.5 × 2.5 × 2.75 mm^3^, interslice gap = 0.25 mm, 310 volumes, 12-min acquisition). Participants were instructed to remain awake with their eyes open. Preprocessing was done using FSL 5 (FMRIB 2012, Oxford, United Kingdom, https://www.fmrib.ox.ac.uk/fsl) and included brain extraction, removal of the first four volumes, motion correction by regressing out six motion parameters, and spatial smoothing at 5-mm full width half maximum. An independent component analysis was performed for automatic removal of motion artifacts (ICA-AROMA) (Pruim et al., 2015), followed by regressing out white matter and cerebrospinal fluid signals and high-pass filtering (100 s cutoff). Mean absolute motion did not exceed 0.6 mm for any participant; the median was 0.27 mm (0.08–0.59 mm). The rsfMRI data were registered to native 3D T1 space using boundary-based registration. The BNA atlas was then reverse-registered to each participant’s functional data using nearest-neighbor interpolation. For every participant, a mask containing only gray matter voxels with reliable rsfMRI signal was constructed by combining a gray matter mask and an rsfMRI mask, excluding all voxels with a signal intensity in the lowest quartile of the robust range (for more details, see Eijlers et al., 2017). Time series were extracted from all atlas regions by averaging time series across all voxels within each region. Thirteen regions with signal loss (i.e., regions with zeros in the functional connectivity matrices) due to magnetic field inhomogeneities in these echo-planar imaging sequences were removed from further analyses across all participants and modalities. Thus, 197 atlas regions remained for all further analyses. Finally, for every participant, Pearson correlation coefficients between all pairs of time series were calculated to obtain a functional connectivity matrix. Correlation coefficients were absolutized, as most network metrics do not take into account negative values, but inverse correlations may carry relevant information (Chai et al., 2012; Zhan et al., 2017).

### Magnetoencephalography

MEG data were recorded in a magnetically shielded room (Vacuumschmelze GmbH, Hanau, Germany) using a 306-channel (102 magnetometers and 204 gradiometers) whole-head MEG system (Elekta Neuromag Oy, Helsinki, Finland) with a sampling frequency of 1250 Hz during a no-task, eyes-closed condition for 5 minutes, an eyes-open condition for 2 minutes, and a final eyes-closed condition for another 5 minutes, with the participant in supine position. Here, we used only the first eyes-closed recording for all further analyses. An anti-aliasing filter of 410 Hz and a high-pass filter of 0.1 Hz were applied online. The cross-validation signal space separation (xSSS) (van Klink et al., 2017) was applied to aid visual inspection of the data. We removed channels containing no signal or noisy signal, with a maximum of 12 channels removed per participant. Further noise removal was performed offline using the temporal extension of signal space separation (tSSS) (Taulu & Simola, 2006) in MaxFilter (version 2.2.15). The head position relative to the MEG sensors was recorded continuously using the signals from five head-localization coils. Coil positions and the scalp outline were digitized using a 3D digitizer (Fastrak, Polhemus, Colchester, VT). A surface-matching procedure was used to achieve co-registration of the participant’s digitized scalp surface and their anatomical MRI, with an estimated resulting accuracy of 4 mm (Whalen et al., 2008). A single best fitting sphere was fitted to the outline of the scalp as obtained from the co-registered MRI, which was used as a volume conductor model for the beamformer approach described below. The co-registered MRI was spatially normalized to a template MRI, and the voxels in the normalized co-registered MRI were again labeled according to the same atlas. We then used a scalar beamforming approach (Hillebrand et al., 2012) to reconstruct the source of neurophysiological activity from the sensor signal. The beamformer weights were based on the lead fields, the broadband (0.5–48 Hz) data covariance, and noise covariance. The data covariance was based on, on average, 298 s of data (range 293–314 s). A unity matrix was used noise covariance. Broadband data were then projected through the normalized beamformer weights to obtain time series for each atlas region. Out of all the voxels that constitute an atlas region, the centroid (Hillebrand et al., 2016) was selected to reconstruct localized MEG activity, resulting in time series for each of the 197 included cortical regions. For all participants, we included the first 88 epochs of 4,096 samples (3.28 s) of the obtained time series (total length 4 min and ∼48 s). Fast Fourier transforms were applied to filter the time series into six frequency bands: delta (0.5–4 Hz), theta (4–8 Hz), lower alpha (8–10 Hz), upper alpha (10–13 Hz), beta (13–30 Hz), and gamma (30–48 Hz). We then computed the phase lag index (PLI) (Stam et al., 2007) between the frequency-filtered time series of all pairs of regions using custom-made scripts in MATLAB (R2018b, Mathworks, Natick, MA) to obtain weighted functional connectivity matrices.

### Single-Layer Network Construction and Analysis

First, we constructed minimum spanning trees (MST) for the six frequency band–specific MEG networks by applying Kruskal’s algorithm (Kruskal, 1956) to the functional connectivity matrices. The MST is a binarized subgraph of the original graph that connects all the nodes in the network without forming loops. This represents the backbone of the network (Stam et al., 2014; Tewarie et al., 2015), which, importantly, is not hindered by common methodological issues such as effects of connection strength or link density on the estimated topological characteristics of networks, while measures calculated on this sparse network are closely related to measures computed on the original underlying network (Tewarie et al., 2015). Edge weights were defined as the inverted PLI values (1/PLI) when constructing the *minimum* spanning tree, since we were interested in the strongest connections (Tewarie et al., 2014).

While the usage of the MST is ubiquitous in MEG studies (Blomsma et al., 2022), it is only rarely used in dMRI and rsfMRI studies, where weighted networks are more commonly analyzed. As we here aimed to compare a relatively new multilayer network approach to the now often-used single-layer methodology, we primarily opted to remain as close as possible to the existing literature on single-layer networks. We therefore calculated nodal eigenvector centrality (EC) individually for each of the six MEG MSTs, and for the fully connected weighted dMRI and rsfMRI connectivity matrices, using the brain connectivity toolbox (https://sites.google.com/site/bctnet/) in MATLAB. EC is a measure of nodal centrality that assumes that a node is more influential if it is connected to nodes that are highly central themselves, and thus considers both the connections of a node itself as well as the connections of its neighbors. This makes it an interesting measure of centrality that takes the entire network into account. Additionally, EC is a spectral measure, and may therefore be less sensitive to noise than other measures of centrality (Kardos et al., 2020), particularly when applied to the backbone of the network. Furthermore, EC has been shown to be highly relevant for cognition in studies using dMRI (Fagerholm et al., 2015), rsfMRI (Eijlers et al., 2017), and MEG (Hardmeier et al., 2012). For a more detailed explanation of the EC and its mathematical definition, see Fornito et al. (2016).

Finally, we extracted and subsequently averaged the ECs of all nodes belonging to the FPN to obtain one value per single-layer network per participant (for a total of eight values per participant), allowing us to assess interindividual differences in the integration of the FPN in the context of the entire brain network. Regions belonging to the FPN were defined based on an earlier categorization (Vriend et al., 2018) of the regions of the BNA according to the classical seven-network parcellation by Yeo et al. (2011).

### Multilayer Network Construction and Analysis

A multiplex network is a multilayer network used to describe different interactions between the same set of nodes (Bianconi, 2018). In this context, each layer is characterized by a different modality of interaction. Therefore, this mathematical framework is useful to encode information from brain networks created using different edge weights or imaging modalities as long as all layers are built using the same atlas. In such a multiplex network, links between different layers, also known as interlayer links, exclusively connect the same node or brain region across layers.

**Figure 2.**
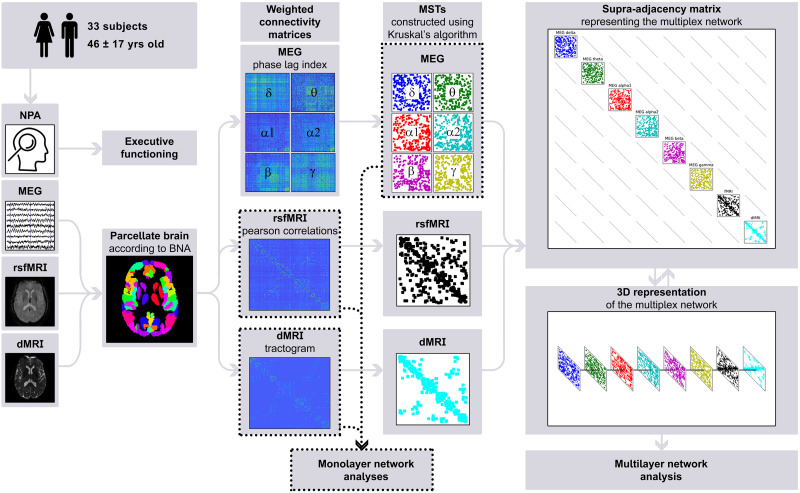
Schematic overview of the analysis pipeline. For every participant, neuropsychological data was used to compute cognition scores; raw imaging data obtained from diffusion MRI, resting-state functional MRI, and magnetoencephalography was preprocessed; the brain was parcellated according to the Brainnetome Atlas; connectivity was calculated to construct weighted connectivity matrices; minimum spanning trees of the weighted matrices were constructed using Kruskal’s algorithm; and finally a supra-adjacency matrix representing a multilayer network was constructed. Note that single-layer network measures were computed on the minimum spanning trees of the magnetoencephalography frequency bands, but weighted data was used for diffusion MRI and resting-state functional MRI. NPA = neuropsychological assessment; MEG = magnetoencephalography; rsfMRI = resting-state functional MRI; dMRI = diffusion MRI; BNA = Brainnetome Atlas; MST = minimum spanning tree.

There is, as of yet, no established method for determining biologically meaningful weighted interlayer links between different modalities. Additionally, network metrics can potentially be biased by differences in link density (especially when comparing dMRI networks, which are inherently sparse, and functional networks) and average connectivity across layers and between participants (Mandke et al., 2018). Here, we therefore decided to construct binary multiplex networks. Consequently, in addition to the MEG MSTs described in the section Single-Layer Network Construction and Analysis, we used Kruskal’s algorithm to construct MSTs for the dMRI and rsfMRI data. We then integrated these eight MSTs to obtain an interconnected multiplex network for every participant. Each participant’s multiplex thus consisted of L = 8 layers (one for dMRI, one for rsfMRI, and one for each of the six MEG frequency bands), with each layer containing the same set of *N* = 197 nodes (atlas regions), and each spanning tree and thus layer having *M* = *N* − 1 = 196 intralayer links. The weights of the interlayer connections were set to 1, identical to the intralayer connections. The resulting multilayer network was represented as an LxN by LxN supra-adjacency matrix with diagonal blocks encoding intralayer connectivity for each modality and off-diagonal blocks encoding interlayer connectivity. Supra-adjacency matrices were then exported to Python (version 3.6, Python Software Foundation, available at https://www.python.org), and multilayer nodal EC was computed according to earlier work. Briefly, we first computed the leading eigenvector of the supra-adjacency matrix, and we then aggregated the corresponding eigenvector of size LxN into a vector of size N, by averaging the entries corresponding to the same nodes, as described in De Domenico et al. (2013). To do so, we used custom-made scripts that integrate the Python libraries multiNetX (Solé-Ribalta et al., 2013) and NetworkX (version 2.3) (Hagberg et al., 2008), which can be found on GitHub (https://github.com/nkoub/multinetx and https://github.com/networkx, respectively). To validate our code, we compared, for a subset of our data, the EC values obtained through the use of our scripts to those obtained using muxViz, a well-known tool for multilayer analysis (De Domenico et al., 2015b). We then again extracted and averaged ECs of the FPN nodes, yielding one value for multilayer EC per participant. A schematic overview of the methods can be found in Figure 2, and a 3D plot of a multilayer network can be found in the Supporting Information. All of the custom-made scripts, as well as the data that we used in this study, can be found on this project’s GitHub page (https://github.com/multinetlab-amsterdam/projects/tree/master/mumo_paper_2021).

### Statistical Analyses

Frequentist statistics were used, with a significance threshold of 0.05 set before starting the analyses, through SPSS (version 26, IBM Corp., Armonk, NY). To assess the relation between single- or multilayer EC of the FPN and age, sex, and education-corrected EF scores, we performed a multiple regression analysis. With EF as the dependent variable, average EC values of the FPN of each of the eight single-layer networks described in section Single-Layer Network Construction and Analysis were added in a first block using a backward stepwise procedure (*F* probability for removal 0.10), and the average EC of the FPN of the multilayer network was entered in a second block. To assess whether these data met the assumption of collinearity, we ran multicollinearity diagnostics based on the variance inflating factor (VIF). The VIF of a predictor is calculated by doing a linear regression of that predictor on all other predictors, after which the VIF is defined by 1/(1 − *R*^2^). A high VIF is thus indicative of the presence of multicollinearity. Tolerance is the inverse of the VIF, and it is suggested that it should not be below 0.2 (Menard, 1995), which we used as a threshold in our manuscript. Regression models were checked for normality of residuals using a Q-Q plot.

### Post Hoc Analyses

To ensure the validity of our main result, we performed several additional post hoc analyses. Firstly, we repeated the multiple regression analysis described above with a forward selection procedure (*F* probability for entry 0.05). Additionally, to explore whether the results were affected by our decision to compute single-layer network metrics on the MSTs for the MEG networks, but on the fully connected weighted networks for the fMRI and dMRI networks, we re-ran the same regression using ECs of the FPN calculated on the MSTs of all the single-layer networks. Also, to confirm the relevance of the multilayer framework, we performed a multiple regression relating the average FPN centrality of all single-layer networks to executive functioning. As before, we added the average FPN centrality values of each of the eight single-layer networks in a first block by using a backward stepwise procedure; we then entered the average EC of the FPN across the single-layer networks in a second block. In order to assess whether EC was, as hypothesized, most sensitive to individual differences in EF, we also computed the degree centrality of the multilayer network and performed a linear regression relating the average multilayer degree centrality of the FPN to executive functioning. Furthermore, we performed a leave-one-out cross-validation for the final regression model of multilayer EC of the FPN and EF to assess whether regression results were not driven by any single subject, and computed the cross-validated coefficient of determination (*R*^2^) using the following equation:

$$R^{2}=100\times \left(1-\frac{\sum_{i=1}^{n}{\left(y_{i}-{\overset{´}{y}}_{i}\right)}^{2}}{\sum_{i=1}^{n}{\left(y_{i}\right)}^{2}}\right),$$

with *n* being the number of measurements, *y* the vector of measurements, and *ý* the vector of model predictions, as described previously (Yeatman et al., 2014). In addition, we repeated our analysis using a different parcellation, namely the automated anatomical labeling (AAL) atlas (Tzourio-Mazoyer et al., 2002), and re-ran the regression model, to compare to the results that were found using the BNA. Finally, as a post hoc analysis, we explored the relationship between multilayer centrality of the FPN and several other cognitive domains, namely, verbal memory (VM), information processing speed (IPS), working memory (WM), and attention. To this end, we performed four separate linear regression analyses with multilayer EC of the FPN as the independent variable and the cognitive domains as the dependent variables. See the Supporting Information for a detailed specification of the cognitive domains.

Additionally, we performed a post hoc analysis to validate the methodology we used for network thresholding within each layer, by comparing our chosen method of the MST to a preliminary analysis inspired by recent developments on network inference (Nakamura et al., 2016). Briefly, in the latter, surrogate data is used to assign each edge in a network a value reflecting its statistical significance, that is, edges with significance below a chosen level are more likely to be “true” edges. Here, we leveraged this methodology as follows: we (a) create an ensemble of 100 null models for each single-layer matrix for every subject in our cohort, by shuffling the edge weights in the original weighted matrix while preserving the degree, weight, and strength distributions (using the function null_model_und_sign.m of the Brain Connectivity Toolbox); (b) perform a nonparametric Kolmogorov–Smirnov test to assess the significance of each original edge in comparison with the correspondent edges in the randomized matrices (using a significance threshold of 0.05); (c) create a new, thresholded matrix, containing only those edges that were significantly different from their randomized counterparts; and (d) compare the edges of the MST matrix with the significance matrix to establish the fraction of significant edges in the MST matrix.

## RESULTS

### Participant Characteristics

Of the 39 included participants, two participants dropped out before completion of the study, two were excluded during the study because of contra-indications for MRI, and another two were excluded after visual inspection of their MRI data revealed artifacts. This resulted in a total of 33 included participants with complete structural MRI, dMRI, rsfMRI, MEG, and neuropsychological data that were used in the analyses. Of these participants, 18 were female and 15 were male. They were well spread out in terms of age, ranging between 22 and 70 years old, with a mean age of 46 ± 17 years. Participants were mainly higher educated.

### Network Correlates of Cognition

**Figure 3.**
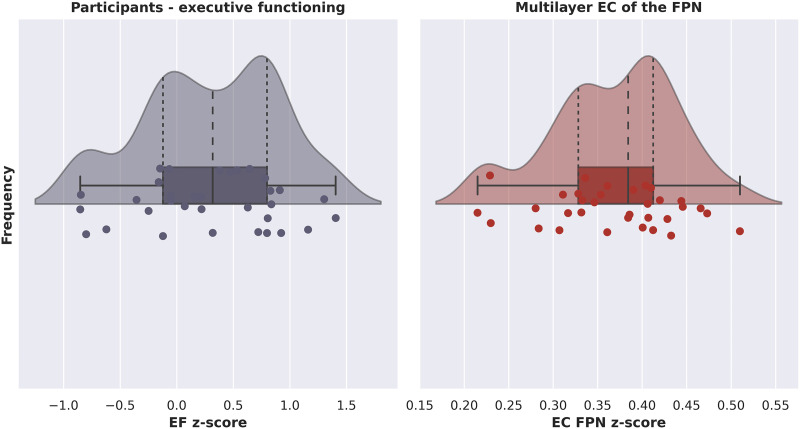
Raincloud plots showing probability density, summary statistics, and individual datapoints of the *z*-score per participant of executive functioning (left) and multilayer eigenvector centrality of the fronto-parietal network (right). EF = executive functioning; EC = eigenvector centrality; FPN = fronto-parietal network.

**Figure 4.**
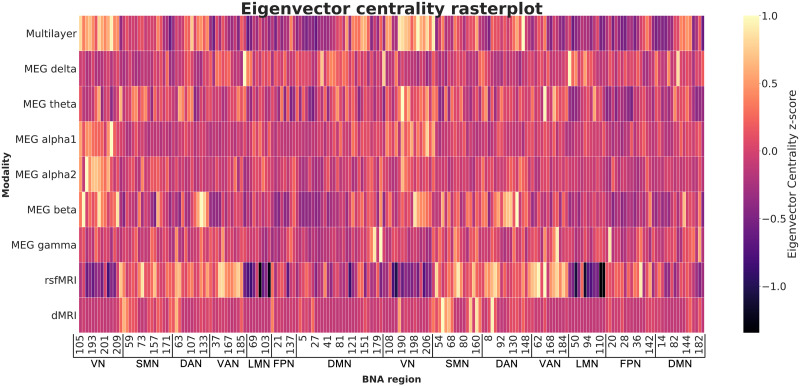
Raster plot showing eigenvector centrality in the eight single-layer networks and the multilayer network averaged over all subjects, ordered by subnetwork (all left-hemisphere regions followed by all right-hemisphere regions). Yellow indicates regions with high EC. This shows the differences in ‘centrality profiles’ across modalities. BNA region numbers refer to the labels as given in Supporting Information Table S1. MEG = magnetoencephalography. rsfMRI = resting-state functional MRI; dMRI = diffusion MRI; BNA = Brainnetome Atlas; VN = visual network; SMN = somatomotor network; DAN = dorsal attention network; VAN = ventral attention network; LMN = limbic network; FPN = fronto-parietal network; DMN = default mode network.

Figure 3 shows a raincloud plot with the distribution of EF *z*-scores for all participants. Importantly, as indicated by the wide range of normed *z*-scores, our sample was diverse in terms of EF performance. There was no evidence of problematic multicollinearity between network variables as indicated by their tolerance values, which were all greater than 0.2 (Menard, 1995). Figure 4 shows exemplar values of EC for the multilayer network as well as all the single-layer networks, averaged over all subjects.

**Figure 5.**
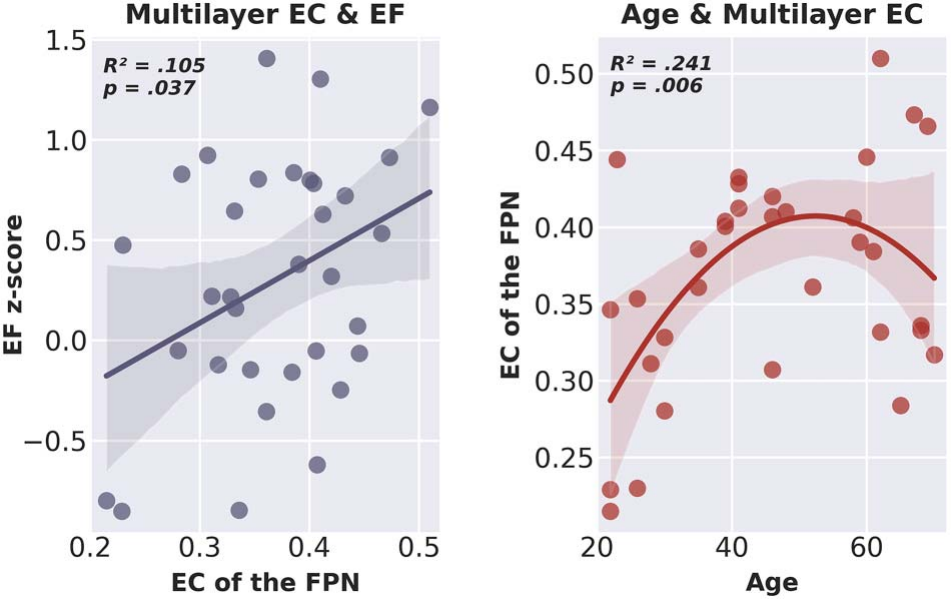
Scatter plot including line of best fit of multilayer eigenvector centrality of the fronto-parietal network and executive functioning (left) and age and multilayer eigenvector centrality of the fronto-parietal network (right). EC = eigenvector centrality; EF = executive functioning; FPN = fronto-parietal network.

Testing our hypotheses, none of the single-layer network eigenvector centralities survived the backward stepwise selection, see Table 1 for the coefficients of the included and excluded variables. The final regression model, containing only multilayer EC of the FPN as a predictor of EF, was statistically significant (*R*^2^ = .133, adjusted *R*^2^ = .105, *F*[1, 31] = 4.753, *p* = .037). There was no significant increase in *R*^2^ from the second-to-last model, containing two predictors (single-layer EC in the lower alpha band and multilayer EC), to this final significant model. These results suggest that only EC of the FPN of the multilayer network was a significant predictor of EF, and that a higher multilayer EC of the FPN was related to better EF (see Figure 5).

**Table 1.** Standardized beta coefficients and *p* values of included and excluded variables of the regression models

**Multilayer EC of the FPN and EF**		
	**β**	***P***
**Final model (*R*^2^_*adj*_ = .105)**		
Multilayer EC	.365	.037*
**Excluded variables**		
EC MEG delta	.047	.798
EC MEG upper alpha	.056	.752
EC dMRI	.074	.669
EC MEG theta	−.065	.716
EC rsfMRI	.097	.587
EC MEG beta	.144	.407
EC MEG gamma	−.204	.234
EC MEG lower alpha	−.238	.163
**Age & multilayer EC of the FPN**		
	**β**	***P***
**Final model (*R*^2^_*adj*_ = .241)**		
Age	.014	.010*
Age squared	.00013	.021*

*Note*. Top: multilayer eigenvector centrality of the fronto-parietal network and executive functioning. Bottom: age and multilayer eigenvector centrality of the fronto-parietal network. EC = eigenvector centrality; FPN = fronto-parietal network; EF = executive functioning; MEG = magnetoencephalography; dMRI = diffusion MRI; rsfMRI = resting-state functional MRI. Asterisk (*) indicates significance at the *p* < 0.05 level.

Post hoc testing confirmed these results. Repeating the analyses using a forward stepwise procedure resulted in an identical, significant final model, containing only multilayer EC as a predictor of EF (*R*^2^ = .133, adjusted *R*^2^ = .105, *F*[1, 31] = 4.753, *p* = .037). The regression using the ECs of the FPN calculated on the MSTs of all single-layer networks (instead of the full matrix for dMRI and rsfMRI) likewise yielded comparable results to the regression model implemented initially, as the final model contained only the multilayer EC of the FPN. The multiple regression using the average EC of the single-layer networks instead of the multilayer EC did not reach significance (*p* = .328 for the final model containing only the average EC of all single-layer networks), indicating that multilayer EC does not simply represent the average of all single-layer ECs. Multilayer degree centrality of the FPN did not relate significantly to EF (*p* = .091), suggesting that, in the context of a multilayer framework, EC is a more sensitive correlate of EF than degree centrality. The leave-one-out cross-validation resulted in a cross-validated coefficient of determination of 20.75 as opposed to 30.22 for the full, non-cross-validated, model. Repeating the analysis using a different atlas resulted in a nonsignificant final model, albeit with comparable explanatory value to the model based on BNA data (*R*^2^ = .177, adjusted *R*^2^ = .122, *F*[2, 32] = 3.220, *p* = .054); however, single-layer dMRI EC of the FPN was a significant individual predictor in the final model (*p* = .021). Finally, additional post hoc analyses revealed associations between multilayer EC of the FPN and other cognitive domains. There were no significant associations between multilayer EC of the FPN and VM (*p* = .227) and attention (*p* = .565); however, there were significant associations between multilayer EC of the FPN and IPS and WM. Higher multilayer EC of the FPN was related to better IPS, *R*^2^ = .395, adjusted *R*^2^ = .129, *F*[1, 31] = 5.744, *p* = .023; β = .395. Similarly, higher multilayer EC of the FPN was related to better WM, *R*^2^ = .171, adjusted *R*^2^ = .144, *F*[1, 31] = 6.402, *p* = .017; β = .414.

### Multilayer Network Correlates of Age

To further validate the relevance of multilayer FPN centrality, we tested its relationship with age. Single-layer network studies have revealed that the brain network tends to become more efficiently integrated in early life (Hwang et al., 2013), after which its development plateaus during middle age (Cao et al., 2014) and subsequently regresses to a less integrative topology with older age (Onoda & Yamaguchi, 2013). This relation is also reflected in changes in for example whole-brain (Hedman et al., 2012) and white matter volume (Ge et al., 2002; Walhovd et al., 2005) across the life-span, indicating that the quadratic relationship between brain measures and age may be a general developmental principle. We therefore hypothesized an inverted-U relation between age and multilayer EC of the FPN. We employed a hierarchical multiple regression model with multilayer centrality as the dependent variable. Age was entered in a first block, and the square of age was added to the model in a second block.

See Figure 3 for a raincloud plot of multilayer centrality, showing the distribution of multilayer network EC of the FPN for all participants. The final model with both age and age squared indicated a statistically significant quadratic relation between age and multilayer EC of the FPN (*R*^2^ = .289, adjusted *R*^2^ = .241, *F*[2, 30] = 6.082, *p* = .006). The square of age added significantly to the model, leading to an increase in *R*^2^ of .140 (*F*[1, 30] = 5.915, *p* = .021), suggesting that the quadratic model indeed more accurately explained age variations than the simple linear model. The coefficients of the included variables are reported in Table 1; Figure 5 shows the relation between age and multilayer EC of the FPN.

### Post Hoc: Edge Inference

The results of the comparison between the individual edges in the MST networks used in our analyses with its correspondent edges in an ensemble of 100 randomized matrices are detailed in Table 2. On average, over all modalities and all subjects, 74.2% of the edges in the MSTs are statistically significant. In contrast, on average only 3.9% of edges in the fully weighted networks are significant. This preliminary analysis provides evidence that most links in our multilayer network are in fact statistically “true” edges.

**Table 2.** Edge inference: Comparison between the mean accuracy of the complete network versus the MST network

**Modality**	**Weighted network**	**MST network**
MEG delta	3.8%	73.5%
MEG theta	3.8%	73.7%
MEG lower alpha	3.8%	69.7%
MEG upper alpha	3.8%	74.1%
MEG beta	3.7%	68.0%
MEG gamma	3.9%	75.1%
rsfMRI	4.9%	73.5%
dMRI	3.6%	85.7%
**Average**	**3.9%**	**74.2%**

*Note*. Accuracy represents percentage of edges where *p* < 0.05. For the full weighted network, the mean accuracy is lower than 5% for all modalities; for the MSTs, the mean accuracy is greater than 67% for all modalities. MST = minimum spanning tree; MEG = magnetoencephalography; rsfMRI = resting-state functional MRI; dMRI = diffusion MRI.

## DISCUSSION

We studied how multilayer centrality of the FPN was related to individual differences in EF, and whether this provided additional information to modality- and frequency-specific single-layer FPN centrality. We found that higher multilayer FPN centrality related to better EF, whereas FPN centrality of single-layer networks did not significantly explain differences in EF between healthy adults. Finally, post hoc analyses established an inverted-U relationship between age and multilayer centrality of the FPN.

Firstly, at least for the multilayer network, these results are in line with other studies displaying the importance of FPN network centrality for EF. The relation between FPN centrality and, by extension, network integration and cognition has been well-established in single-layer networks, using different neuroimaging and neurophysiological modalities. Increased integration of the FPN within the entire brain network specifically has been related to better EF in studies utilizing dMRI (Caeyenberghs et al., 2016), rsfMRI (Cole et al., 2012; Takeuchi et al., 2015), and MEG (van Dellen et al., 2013). While network segregation is thought to enable fast processing of lower order information (e.g., analysis of visual inputs) (Clune et al., 2013), highly central nodes like those within the FPN facilitate global communication between these segregated communities, presumably enabling higher order cognitive processes and specifically EF (see, e.g., Bertolero et al., 2017; Sporns, 2013). Our significant results for EC, but not degree centrality, may indicate that EC, which is a spectral measure, may be optimally sensitive to individual differences in EF. Moreover, the associations we found between multilayer centrality of the FPN and several other cognitive domains, namely, IPS and WM, suggest that the importance of FPN integration is not exclusive to EF as defined in this study. IPS, WM, and EF are closely related constructs (see, e.g., McCabe et al., 2010), and the FPN may play a similar integrative role in these aspects of cognition.

Secondly, our results demonstrate the relevance of multimodal network analysis through a multilayer network approach in explaining cognitive variance. While FPN centrality of the unimodal networks did not relate significantly to EF, higher FPN centrality of the multilayer networks was indeed associated with better EF. Visual exploration of our data confirmed that the level of integration per node depends on the modality on which the network is based, and that this is again different for the multilayer network. Central nodes (i.e., nodes with high EC) in the multilayer network are thus not the same as central nodes in the single-layer networks (see Figure 4). Other multilayer studies have similarly reported that the precise node that can be considered most central in a single-layer network may not serve as the most central node in a multilayer network, and vice versa (De Domenico et al., 2016; Yu et al., 2017). We build upon these studies by demonstrating that multimodal information captures variance in EF that networks obtained from a single modality do not.

Finally, the quadratic relation between age and multilayer centrality possibly reflects the rise and decline of brain network efficiency across the life-span and is in line with findings from studies reporting on other brain-related processes as well as unimodal network data. Whole-brain volume has been shown to increase up to about 35 years of age, and then decline at a slow but steady rate (Hedman et al., 2012). Another study reported that the percentage of white matter volume (relative to intracranial volume) slowly increases until approximately 40 years of age, after which it decreases quickly (Ge et al., 2002). Network measures have likewise been shown to be sensitive to age effects. Single-layer brain networks become more segregated or modular during development (He et al., 2019), and connectivity of highly central regions increases from childhood to adulthood (Hwang et al., 2013), suggesting that the brain network becomes increasingly efficient (i.e., a better balance between segregation and integration emerges) with maturation. However, after a certain age, modularity of the network seems to decrease (Onoda & Yamaguchi, 2013), indicating a degradation of the efficiency of the brain network. In our study, a similar plateauing seems to occur at middle age. Together with the literature, these findings indicate that brain characteristics have a universal tendency to develop in a particular manner across the life-span. As such, FPN centrality of the multilayer network may be considered an age-relevant metric in future studies. Note that we corrected EF scores for age, such that the association we find between EF and multilayer centrality cannot be ascribed to age effects alone. However, future work is needed to disentangle the exact relationship between age, network centrality, and EF: it is possible that age impacts both EF and network measures, necessitating future studies either investigating the relationship between EF and network centrality in a homogeneously aged sample, or much larger studies able to regress age effects from both EF and network measures.

The biological interpretation of the multilayer network used in this work deserves further consideration. Importantly, the spatial definition of nodes is identical across layers: the nodes in each modality represented the same brain regions. The use of the Brainnetome Atlas, which is based on both structural and functional connectivity pattern similarity within and across brain regions, supports the assumption that these nodes can indeed be seen as canonical units across layers. This is further evidenced by the results of repeating the analysis using a different atlas: the final model did not reach statistical significance, although the explanatory value was comparable to the results based on the Brainnetome Atlas, and FPN centrality of the single-layer dMRI network was a significant individual predictor. Indeed, single-layer reviews have shown that network metrics are not necessarily robust across parcellations, especially when the number of regions differs (Arslan et al., 2018; de Reus & van den Heuvel, 2013). The AAL atlas is a purely anatomically driven parcellation that comprises 78 cortical regions as opposed to 210 regions in the Brainnetome Atlas and is based on the single-subject MNI image (Tzourio-Mazoyer et al., 2002), which may thus be less suited to multilayer analyses, especially when using multimodal connectivity data as was the case here. We then used interlayer links between the same brain regions (nodes) across layers to integrate different modalities. The biological assumption here is that structure and function conflate maximally within the same brain region. There is ample evidence that this assumption holds across macroscopic modalities when correlating, for instance, structural and rsfMRI connectivity patterns across the whole brain (Garcés et al., 2016; Honey et al., 2010; Park & Friston, 2013). The spatial variation that exists in nodal correlations between structural and functional connectivity (Honey et al., 2009; Vázquez-Rodríguez et al., 2019), however, may indicate that although this connectivity is highest within the same region instead of between regions, the linkage between layers varies per region. Such variations were not taken into account in the current work, where we used MSTs of the individual layers for the construction of multiplex networks and set the weights of all interlayer connections in the multilayer networks to one.

It should be noted that using the MST results in networks of very low density (∼1% for typical brain network sizes). Results may not be the same at different densities, and the effect of thresholding at different densities perhaps warrants future exploration. Nevertheless, our decision to use the MST as the foundation for building the multilayer networks in the present study was well considered. Network thresholding, although well explored in isolated modalities, has not yet been studied extensively in multilayer networks. What we do know, however, is that the same methodological issues that apply to single-layer networks (i.e., network measures are influenced by differences in link density and average connectivity) also apply to multilayer networks, and that constructing MSTs of the individual layers of a multilayer network can adequately correct for these biases (Mandke et al., 2018). Furthermore, replicability of results across thresholding densities is an inherent issue of all methodologies for network thresholding (e.g., Buchanan et al., 2020; Garrison et al., 2015). The MST has not only been amply used in studies investigating the cognitive relevance of particularly MEG single-layer network topology for cognition, thus providing a logical springboard for more complicated analyses such as the multilayer framework we employed here; it is also considered the backbone of the network (Stam et al., 2014; Tewarie et al., 2015), using mainly the original network’s strongest connections, which are unlikely to be purely driven by noise. Indeed, the initial post hoc analyses we performed give a strong indication that most of the edges included in each network by the MST algorithm are statistically significant. Future work, though, may benefit from a more data-driven approach for network reconstruction based on statistical sampling of network ensembles, to ensure that networks comprise statistically significant edges (Raimondo & De Domenico, 2021).

Next, future studies may attempt to incorporate weighted interlayer links to represent the spatial variation in within-region correlations across modalities. Additionally, even when choosing to assign the same weight to all interlayer links, other multilayer work has suggested that an optimal value for the interlayer weight exists (De Domenico et al., 2016), and future implementations of the multilayer framework could benefit from optimization of the interlayer weights. Another potential shortcoming of the binarization of link weights is that it eliminates layer dominance (Sahneh & Scoglio, 2014): some layers may have a stronger influence on multilayer network characteristics than others, but when all layers carry the same importance, this information is lost. However, just as some layers may drive the properties of the multilayer more strongly than other layers, other layers may play a negligible role. This raises the question whether all possible layers should be included, or whether an a priori selection should be made, and, if so, how this selection should be made. A few papers have begun to probe this issue, for example, in brain networks (De Domenico et al., 2015a) and in a dataset of social contacts (Casiraghi, 2017), but the layer selection problem in multimodal brain networks warrants further exploration. Furthermore, although interlayer connectivity may be maximal within brain regions, there is potential connectivity between different regions across different modalities, that is, cross-talk between node A in modality X and node B in modality Y may be relevant to overall functioning of the network. A general multilayer network formulation allows interlayer links between all nodes in all layers (see, e.g., Bianconi, 2018). However, multimodal datasets present considerable challenges when constructing a full multilayer network. Chief among them is determining biologically meaningful interlayer links between different modalities at the individual participant level. Additionally, a recent modeling study revealed that interlayer connectivity is driven mostly by one-to-one (i.e., multiplex) connections (Tewarie et al., 2021), and as evidenced by previous empirical studies (De Domenico et al., 2016; Yu et al., 2017), a multiplex approach is therefore a logical and intuitive first step for analyzing multidimensional data. Lastly, we defined the FPN based on a predefined classification. As such, the FPN was comprised of the same regions across the different single-layers as well as in the multilayer network. However, subnetworks like the FPN and the hubs within them have been found to vary depending on the modality used (Brookes et al., 2011; Garcés et al., 2016), and have also been shown to be different in a multiplex compared to a single-layer network (De Domenico et al., 2016). Additionally, there is a large individual variability in the functional topography of the FPN (Marek & Dosenbach, 2018). A more data-driven approach to the formulation of the FPN may therefore further increase the explanatory power of the multilayer approach.

Some additional limitations need to be taken into consideration. In this study, we chose to absolutize negative correlations in the fMRI networks, but it should be noted that there is an ongoing debate on what negative correlations indicate and how to best deal with them (Hallquist & Hillary, 2018). However, the relative magnitude of such negative edge weights is low and the MST only incorporates the strongest links, and as such it is unlikely that this decision has impacted our results. Furthermore, there are some inherent drawbacks to the stepwise regression models we employed to answer our research question. Although this approach was the one best suited to answer our research question, it is imperative that our work is replicated in larger samples using our work to establish more straightforward regression models. Indeed, the relatively small sample size of the present study is particularly important to take into account: the absence of any single-layer effects could be due to a lack of statistical power, rather than the true absence of any correlations between single-layer network metrics and cognition. Our sample was powered using previous studies correlating cognition with single-layer network properties (Douw et al., 2016; Hassan et al., 2017; Lord et al., 2017; Ystad et al., 2011), but these may have overestimated the size of the actual effect: there is significant discussion on the minimal sample size needed to establish reliable brain-behavior associations (Marek et al., 2022). Moreover, since the multilayer approach has not been used to explain cognitive variance before, we could not power for significance of change going from single-layer to multilayer correlates of EF. There was no significant increase in *R*^2^ from the model containing a nonsignificant single-layer plus the multilayer predictor and the model containing only the significant multilayer predictor. We therefore cannot claim, statistically, that the multilayer network approach is a better predictor of EF than the single-layer network approach. However, the fact that the final model containing only the multilayer predictor was significant, whereas the single-layer predictors were not, suggests that (a) sample size may have contributed to a lack of statistical power to find evidence of significant change between these models and (b) a multilayer approach may require less data points in order to observe significant correlates of EF in the brain. We therefore conclude that the multilayer methodology is a potentially valuable framework for explaining EF, although its statistical superiority over single-layer approaches must be assessed in adequately powered, larger, samples.

## CONCLUSIONS

Integration of multimodal brain networks through a multilayer framework relates to EF in healthy adults, and corroborates known brain associations with aging. These findings underline the relevance of a multimodal view on integration of the brain network as a correlate of EF. Furthermore, the multilayer approach may be of particular interest in populations where network alterations differ across modalities. For instance, early neurodegeneration and structural network deterioration of particularly the most central regions in the brain are initially associated with increases in functional communication, after which the functional brain networks seems to collapse (Hillary et al., 2015; Schoonheim et al., 2015). Multilayer network analysis may advance our understanding of the interplay between structural and different functional network aspects in such clinical populations.

## ACKNOWLEDGMENTS

The authors would like to thank the Amsterdam Neuroscience research institute for supporting this study. We would also like to thank Hersenonderzoek.nl, a Dutch online registry that facilitates participant recruitment for neuroscience studies (www.hersenonderzoek.nl). Hersenonderzoek.nl is funded by ZonMw-Memorabel (project no. 73305095003), a project in the context of the Dutch Deltaplan Dementie, Gieskes-Strijbis Foundation, the Alzheimer’s Society in the Netherlands and Brain Foundation Netherlands. We thank the laboratory technicians of the Amsterdam UMC, Department of Clinical Neurophysiology and MEG Center, as well as the scan assistants of the Spinoza Centre for Neuroimaging, for their help with the data acquisition. Finally, we thank all participants for their participation.

## SUPPORTING INFORMATION

Supporting information for this article is available at https://doi.org/10.1162/netn_a_00284 and https://github.com/multinetlab-amsterdam/projects/tree/master/mumo_paper_2021. Additionally, this article made use of scripts available at https://github.com/nkoub/multinetx and https://github.com/networkx.

## AUTHOR CONTRIBUTIONS

Lucas C. Breedt: Data curation; Formal analysis; Investigation; Methodology; Project administration; Software; Visualization; Writing – original draft; Writing – review & editing. Fernando A. N. Santos: Data curation; Formal analysis; Methodology; Software; Supervision; Visualization; Writing – original draft; Writing – review & editing. Arjan Hillebrand: Conceptualization; Formal analysis; Funding acquisition; Software; Writing – review & editing. Liesbeth Reneman: Conceptualization; Funding acquisition; Writing – review & editing. Anne-Fleur van Rootselaar: Conceptualization; Funding acquisition; Writing – review & editing. Menno M. Schoonheim: Conceptualization; Funding acquisition; Writing – review & editing. Cornelis J. Stam: Conceptualization; Funding acquisition; Writing – review & editing. Anouk Ticheler: Investigation; Project administration; Writing – review & editing. Betty M. Tijms: Conceptualization; Funding acquisition; Writing – review & editing. Dick J. Veltman: Conceptualization; Funding acquisition; Writing – review & editing. Chris Vriend: Conceptualization; Formal analysis; Funding acquisition; Methodology; Writing – review & editing. Margot J. Wagenmakers: Investigation; Project administration; Writing – review & editing. Guido A. van Wingen: Conceptualization; Funding acquisition; Writing – review & editing. Jeroen J. G. Geurts: Conceptualization; Funding acquisition; Supervision; Writing – review & editing. Anouk Schrantee: Conceptualization; Funding acquisition; Methodology; Project administration; Supervision; Writing – review & editing. Linda Douw: Conceptualization; Funding acquisition; Methodology; Project administration; Supervision; Writing – original draft; Writing – review & editing.

## FUNDING INFORMATION

Linda Douw, Amsterdam Neuroscience Alliance Grant. Linda Douw, Netherlands Organization for Scientific Research (NWO) Vidi grant, Award ID: 198.015.

## TECHNICAL TERMS

Unimodal: Consisting of a single form of imaging, e.g., only MRI or only MEG.
Multimodal: Consisting of more than one form of imaging, e.g., MRI as well as MEG.
Multilayer network: Network comprised of multiple layers whose nodes interact. Each layer contains different information.
Phase lag index: Measure of connectivity in MEG (or EEG) based on the asymmetry of the distribution of phase differences between two signals.
Multiplex network: Special case of multilayer networks, with interlayer connections present only between a node’s counterparts across layers.
Interlayer links: Connections between two different layers of a multilayer network.
Intralayer links: Connections within one layer (of a multilayer network).
Supra-adjacency matrix: Matrix representation of a multilayer network. Diagonal blocks contain connectivity within a layer; off-diagonal blocks contain connectivity between layers.

## Supporting Information

https://github.com/nkoub/multinetx

https://github.com/networkx

https://github.com/multinetlab-amsterdam/projects/tree/master/mumo_paper_2021
