# Supplementary material for: Multimodal multilayer network centrality relates to executive functioning
Source: Netw Neurosci. 2023 Jan 1;7(1):299–321. doi: 10.1162/netn_a_00284 (PMC10275212; doi:10.1162/netn_a_00284)
Supplement: Supplementary file 1 [file netn-7-1-299-s001.pdf]

Breedt, L. C., Santos, F. A. N., Hillebrand, A., Reneman, L., van rootselaar, A., Schoonheim, M. M., Stam, C. J., Ticheler, A., Tijms, B. M., Veltman, D. J., Vriend, C., Wagenmakers, M. J., van Wingen, G. A., Geurts, J. J. G., Schrantee, A. & Douw, L. (2022). Supporting information for “Multimodal multilayer network centrality relates to executive functioning.”*Network Neuroscience*. Advance publication.
https://doi.org/10.1162/netn_a_00284

Supplementary Materials

Multimodal multilayer network centrality relates to executive functioning

Lucas C. Breedt, Fernando A. N. Santos, Arjan Hillebrand, Liesbeth Reneman, Anne-Fleur van Rootselaar, Menno M. Schoonheim, Cornelis J. Stam, Anouk Ticheler, Betty M. Tijms, Dick J. Veltman, Chris Vriend, Margot J. Wagenmakers, Guido A. van Wingen, Jeroen J. G. Geurts, Anouk Schrantee, and Linda Douw

Network correlates of cognition

To assess the specificity of the reported association between multilayer centrality of the FPN and executive functioning, we performed several *post-hoc* analyses to test the relationship between multilayer centrality of the FPN and several other cognitive domains, namely, verbal memory (VM), information processing speed (IPS), working memory (WM), and attention.

Neuropsychological evaluation

We used validated norms to obtain z-scores of the Dutch version of Rey’s Auditory Verbal Learning Test (RAVLT), the 15 Words Test (15WT; (Van den Burg et al., 1985)); the Memory Comparison Test (MCT; (Van Der Elst et al., 2007)); and the Letter-Digit Modalities Test (LDMT; (Van der Elst et al., 2006)). Briefly, for the 15WT the subject is asked to memorize and recall a list of 15 random items five times consecutively. After a 20 minute delay, the subject is asked to recall the list once more, and to recognize those items from a longer list of 30 words. For the MCT, the subject is asked to memorize a target, and is then presented with a sheet containing multiple rows of letters among which the target occurs multiple times. These need to be crossed out as quickly as possible. The MCT consists of one practice trial, during

which the target is a percentage sign, and four trials of increasing difficulty, during which the target consists of one, two, three, and four letters, respectively. For the LDMT, the subject is shown a substitution key of how the numbers one through nine correspond to nine letters, and is then given 90 seconds to match as many numbers to letters as possible. The test consists of a verbal and a written trial. Raw scores were transformed into z-scores relative to a comparable healthy population. Construction of cognitive domains was based on previous work where Principal Component Analysis was performed to assign individual tests to cognitive domains (Douw et al., 2009; Klein et al., 2003). VM was thus defined as the average of z-scores for 15WT-total (total number of correctly remembered words over five consecutive trials) and 15WT-recall (total number correctly recalled after 20-minute delay). IPS was defined as the average of z-scores for CST-A (time to complete card A), Stroop-I (time to complete card 1), and LDMT-90w (total number of correctly written matches after 90 seconds). WM was defined as the average of z-scores for CST-shift, MCT-intercept (time to complete trial 1), and MCT-slope (time to complete trial 4 minus intercept, divided by 3). Attention was defined as the average of z-scores for CST B (time to complete card B) and Stroop-II (time to complete card 2).

Supplementary Table 1

Brainnetome atlas regions comprising the FPN, including anatomical label. The following regions were excluded from analyses: 47, 48, 69, 70, 94, 101, 111, 113, 115-119.

**BNA region**	**Gyrus**	**BNA label**	**Subnetwork**
177/178	Cingulate Gyrus	A24rv, rostroventral area 24	FPN
29/30	Inferior Frontal Gyrus	A44d, dorsal area 44	FPN
31/32	Inferior Frontal Gyrus	IFS, inferior frontal sulcus	FPN
137/138	Inferior Parietal Lobule	A39rd, rostrodorsal area 39 (Hip3)	FPN

99/100	Inferior Temporal Gyrus	A20cl, caudolateral of area 20	FPN
17/18	Middle Frontal Gyrus	IFJ, inferior frontal junction	FPN
19/20	Middle Frontal Gyrus	A46, area 46	FPN
21/22	Middle Frontal Gyrus	A9/46v, ventral area 9/46	FPN
147/148	Precuneus	A7m, medial area 7 (PEp)	FPN

References

Douw, L., Klein, M., Fagel, S. S., van den Heuvel, J., Taphoorn, M. J., Aaronson, N. K., Postma, T. J., Vandertop, W. P., Mooij, J. J., & Boerman, R. H. (2009). Cognitive and radiological effects of radiotherapy in patients with low-grade glioma: long-term follow-up. *The Lancet Neurology, 8*(9), 810-818.

Klein, M., Engelberts, N. H., van der Ploeg, H. M., Kasteleijn-Nolst Trenité, D. G., Aaronson, N. K., Taphoorn, M. J., Baaijen, H., Vandertop, W. P., Muller, M., & Postma, T. J. (2003). Epilepsy in low-grade gliomas: The impact on cognitive function and quality of life. *Annals of Neurology: Official Journal of the American Neurological Association and the Child Neurology Society*, *54*(4), 514-520.

Van den Burg, W., Saan, R., & Deelman, B. (1985). 15-Woordentest: Provisional Manual. *Groningen: University Hospital, Department of Neuropsychology*.

Van der Elst, W., van Boxtel, M. P., van Breukelen, G. J., & Jolles, J. (2006). The Letter Digit Substitution Test: normative data for 1,858 healthy participants aged 24–81 from the Maastricht Aging Study (MAAS): influence of age, education, and sex. *Journal of clinical and experimental neuropsychology*, *28*(6), 998-1009.

Van Der Elst, W., Van Boxtel, M. P., Van Breukelen, G. J., & Jolles, J. (2007). Assessment of information processing in working memory in applied settings: the paper & pencil memory scanning test. *Psychological medicine*, *37*(9), 1335-1344.
